# Ultrasound assessment of preoperative gastric volume in fasted diabetic surgical patients: A prospective observational cohort study on the effects of glucagon-like peptide-1 agonists on gastric emptying

**DOI:** 10.1016/j.jclinane.2025.111853

**Published:** 2025-05-04

**Authors:** Christopher D. Wolla, Travis J. Pecha, Joel M. Sirianni, Lexi M. Schorg, Bethany J. Wolf, Sylvia H. Wilson

**Affiliations:** a Department of Anesthesiology and Perioperative Medicine, Medical University of South Carolina, 167 Ashley Avenue, Suite 301, Charleston, SC 29425, USA; b Department of Public Health Sciences, Medical University of South Carolina, 167 Ashley Avenue, Suite 301, Charleston, SC 29425, USA

## Abstract

**Background:**

Preoperative gastric ultrasound allows non-invasive qualitative and quantitative assessment of gastric contents aiding in preoperative risk assessment. We hypothesized that appropriately fasted diabetic surgical patients taking GLP-1 agonists would have higher gastric volumes than those not taking GLP-1 agonists.

**Methods:**

This prospective, observational cohort study enrolled diabetic patients undergoing elective surgery, comparing those taking (*n* = 106) and not taking (*n* = 100) GLP-1 agonists. The primary outcome was gastric volume assessed via gastric ultrasound in the right lateral decubitus position. Secondary outcomes included presence of a full stomach (solids/thick liquids or greater than 1.5 mL/kg clear liquid), need for surgery delay, Perlas grade, and occurrence of intraoperative aspiration. The impact of GLP-1 agonist type, duration of use, and timing of last dose on gastric volume was also examined.

**Results:**

Diabetic patients on GLP-1 agonists had significantly higher median gastric volumes compared to patients not on GLP-1 agonists (0.61 mL/kg vs 0.16 mL/kg, *P* < 0.001) and increased odds of a full stomach (OR 11.3, 95 % CI 5.2–24.7, *P* < 0.0001). GLP-1 agonist use correlated with higher Perlas grades (*P* < 0.001). Gastric volumes were significantly higher with GLP-1 agonist use within 7 days of surgery relative to use within 7–14 days or more than 14 days from surgery (*P* < 0.001 for both comparisons).

**Conclusions:**

GLP-1 agonist therapy was associated with higher residual gastric volumes and higher risks of full stomachs in fasted diabetic patients. GLP-1 agonist use within 7 days of surgery was also associated with higher gastric volumes relative to holding therapy for over 7 days, supporting current consensus-based guidelines.

## 1. Introduction

Glucagon-like peptide-1 (GLP-1) agonists have surged in popularity as a treatment for type II diabetes mellitus. Diabetes mellitus is a known cause of delayed gastric emptying with up to 50 % of diabetic patients having symptoms of diabetic gastroparesis which is caused by a combination of autonomic neuropathy, hyperglycemia, and enteric neuromuscular inflammation [1]. In addition to regulating post-prandial glucose levels, these drugs act to delay gastric motility, leading to early satiety and growing popularity for obesity treatment and weight loss [2–4]. Unfortunately, this has coincided with several reports of retained gastric contents and concerns for an elevated risk of perioperative aspiration [5–10]. While the current evidence is yet to elucidate the full safety profile of these drugs in the perioperative period, initial American Society of Anesthesiologist (ASA) consensus guidelines recommend weekly GLP-1 agonist injections be held for one week prior to elective surgery, however newly published multisociety guidances and expert opinions emphasize a shared decision-making process that allows for GLP-1 agonist therapy to be continued preoperatively highlighting individualized risk-mitigating approaches [11–15]. Thus, gathering more information on the impact of GLP-1 agonists on residual gastric contents is critical.

Gastric ultrasound has emerged as a validated assessment modality in measuring gastric volume and contents. This qualitative and quantitative assessment tool enables the anesthesiologist to calculate residual gastric volume and risk stratify patients based on aspiration risk, which can impact perioperative planning [16,17].

The purpose of this prospective observational study was to investigate the difference in gastric volume in diabetic patients taking and not taking GLP-1 agonists after appropriately fasting for surgical procedures. We hypothesized that patients taking a GLP-1 agonist would have increased gastric volumes relative to patients not on GLP-1 agonist therapy.

## 2. Methods

This single center, prospective, observational cohort trial was conducted in accordance with the original protocol following institutional IRB approval (Pro00128783) and written informed consent was obtained from all subjects participating in the trial. The trial was registered prior to patient enrollment at clinicaltrials.gov (NCT05854979; Principal investigator: Christopher Wolla, Date of registration: May 11, 2023). This manuscript adheres to applicable Strengthening the Reporting of Observation Studies in Epidemiology (STROBE) reporting guidelines.

On the day of surgery, diabetic patients were invited to participate, provided with informed consent, and enrolled if eligible by study staff. Inclusion criteria consisted of age > 18 years, scheduled for an elective procedure with general anesthesia or monitored anesthesia care, appropriate fasting per ASA 2017 Fasting Guidelines [18], and diagnosed with diabetes (defined as a diagnosis listed on the medical record, Hb A1C greater than or equal to 6.5 %, fasting blood glucose greater than or equal to 126 mg/dL, and/or on medical treatment for diabetes). Exclusion criteria included body mass index (BMI) greater than 40 kg/m^2^, prior gastric or esophageal surgery, known abnormal or altered gastric anatomy, unable to position for gastric ultrasound, pregnancy, unable to speak and/or read English (due to limited interpreter services), and unable or unwilling to provide informed consent.

After completion of informed consent and review of inclusion/exclusion criteria, subjects were assigned an enrollment number. A trained study team member then collected information on use of a GLP-1 agonist (yes/no), demographic data (age, height, weight, gender, race, ethnicity), pertinent medical history (diabetes type and duration, gastroparesis, chronic kidney disease), and symptom evaluation (nausea and vomiting, abdominal bloating, abdominal pain). For subjects on GLP-1 agonists, the type of medication, duration of therapy, and last administration were recorded.

### 2.1. Gastric ultrasounds

**Fig. 1.**
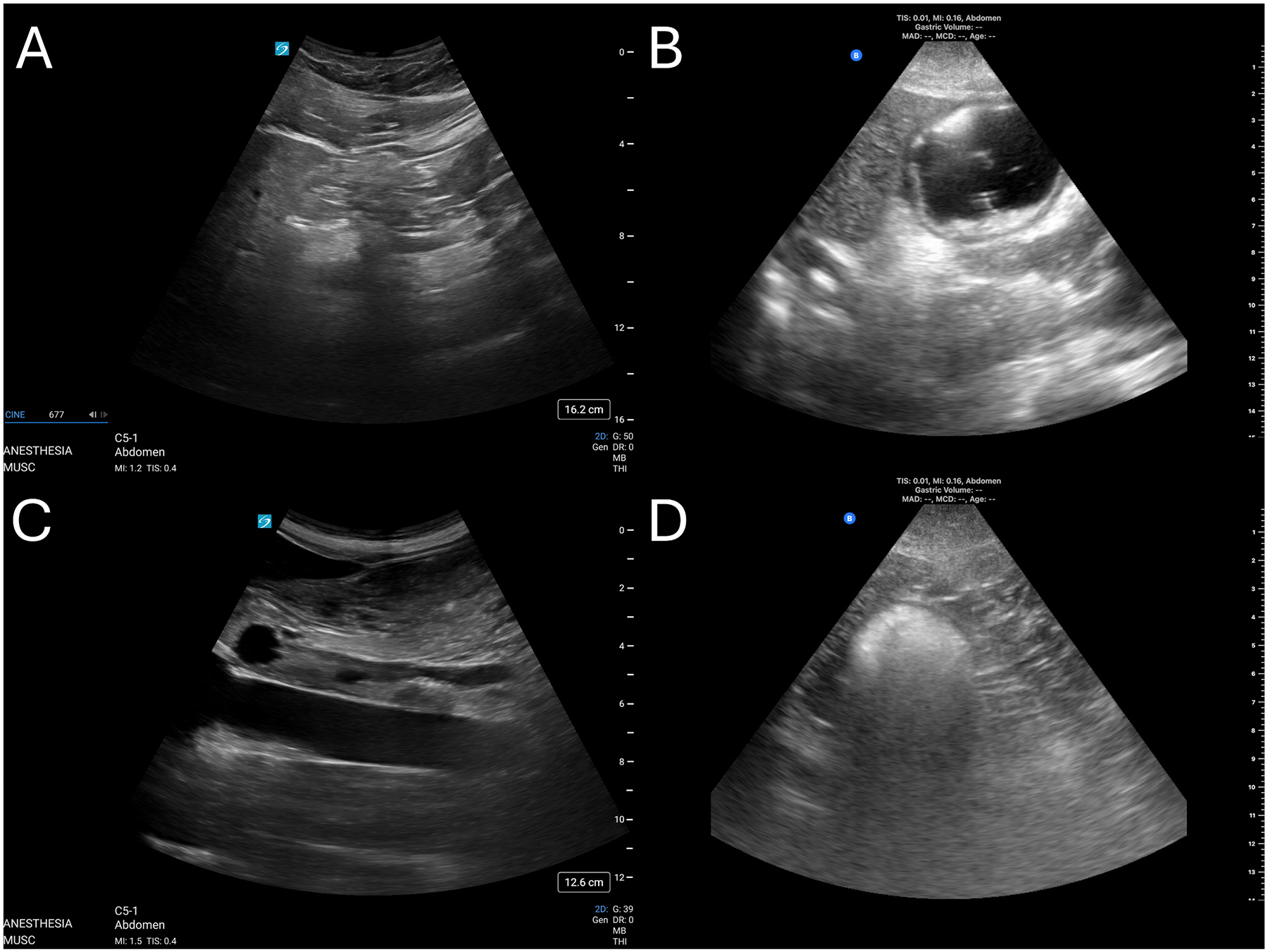
Ultrasound images of qualitative antral appearances in the right lateral decubitus position. (A) Empty antrum, fully collapsed. (B) Distended antrum containing clear liquids. (C) Greatly distended antrum containing thick liquids/late stage solids. (D) Antrum with recent ingestion of solids (“frosted glass” appearance).

All sonography was performed by an attending physician with fellowship training and/or POCUS certification, who also serve as core faculty for anesthesia perioperative ultrasound resident education. Over 70 % of all ultrasounds were completed by authors CDW, TJP, and SHW, and 100 % of ultrasound images and measurements were retrospectively reviewed by authors CDW and TJP. Gastric ultrasound was conducted as previously described [19]. In preoperative holding, subjects were placed in the supine position. A curvilinear, low frequency (5–2 MHz) ultra-sound probe was placed in the sagittal orientation below the xiphoid process and moved along the costal margin to the right until the gastric antrum was identified when located between the left lobe of the liver and the pancreas at the level of the abdominal aorta. The sonographer then identified if the gastric antrum appeared to be empty (yes/no) or contain liquid or solids contents. The patient was then placed in the right lateral decubitus position to measure the cross-sectional area (CSA) of the gastric antrum, the above procedure repeated, and an image was captured. Examples of study images are seen in Fig. 1. Gastric volume (GV) in mL was then calculated using the formula GV = 27.0 + (14.6*CSA)-(1.28*age). Perlas grade was also assigned (0: empty stomach; 1: fluid visible in right lateral decubitus only, suggesting low gastric volume; 2: fluids visible in supine and right lateral decubitus, suggesting high gastric volume) [20]. A full stomach was defined as the presence of solids or gastric volume greater than 1.5 mL/kg. The anesthesia and surgical teams caring for the patient were alerted if the patient was shown to have a gastric volume greater than 1.5 mL/kg, Perlas grade 2, or concern for thick fluids or solid foods to further assess the patient’s risk for aspiration and decided if it was safe to proceed with the elective surgery. All ultrasound data and measurements were later reviewed and confirmed by authors CW or TP.

### 2.2. Outcomes

The primary outcome was gastric volume prior to surgery measured by ultrasound in the right lateral decubitus position in diabetic patients taking and not taking GLP-1 agonists. Additional collected data for all subjects included demographic information, focused medical history, fasting duration, presence of a full stomach on gastric ultrasound, Perlas grade on gastric ultrasound, need for surgery rescheduling or delay, and occurrence of intraoperative aspiration. Demographic data collected included age, height, weight, and self-reported sex, race, and ethnicity. Focused medical history included diabetic type and history (gastroparesis, chronic kidney disease) or symptoms (severe nausea and vomiting, abdominal bloating, abdominal pain) for delayed gastric emptying. In patients taking GLP-1 agonists, type of GLP-1 agonist prescribed, duration of GLP-1 agonist use, and time from last dose were recorded.

### 2.3. Power

As a prior study [21] found that fasted diabetic patients undergoing elective surgery had a mean gastric volume of 57.2 ± 60.5 mL, a sample size of 95 per group was calculated to provide 80 % power to detect a 25 mL increase in volume using a 2-sided test at significance level α = 0.05 and assuming a mean volume in the control group (diabetics not taking GLP-1 agonists) would be similar. Sample was increased to 105 per group (210 total) to account for up to 10 % attrition and allow a sufficient sample size to adjust for up 20 potential confounders.

### 2.4. Statistical analysis

Descriptive statistics were calculated for patient, clinical, and procedural measures. Differences in patient characteristics for categorical data were evaluated using chi-square tests of Fisher’s exact test, 2-sample *t*-tests, or Wilcoxon rank sum tests where appropriate.

The primary outcome of interest was difference in gastric volumes between diabetic patients taking versus not taking GLP-1 agonists. As gastric volume was calculated as GV = 27.0 + (14.6*CSA) −(1.28*age), 0 mL was utilized for any negative measurements. The univariate difference in gastric volume by use of a GLP-1 agonist was evaluated using a Wilcoxon rank sum test. A multivariable propensity weighted logistic-log-normal mixed model of gastric volume was also fit to examine the impact of GLP-1 agonist use controlling for other relevant factors. Propensity weights address heterogeneity between participants by GLP-1 agonist use and were calculated using inverse probability treatment weighting from a logistic regression model including patient (age, biologic sex, self-reported race, and BMI) and clinical characteristics (duration of diabetes and NPO time). This logistic-log-normal mixed model was chosen because of the large number of subjects with a calculated gastric volume of zero. Additionally, this approach accounts for clustering within ultrasounds collected by the same physician. It estimates the probability a participant has a non-zero volume, and conditional on having non-zero volume estimates the volume. For both the logistic and conditional log-normal model, fixed effects for patient age, BMI, and NPO time and a random effect for physician were also included. The natural log of gastric volume for those with some stomach content was used to meet model assumptions.

Additional outcomes examined included whether patients had full stomachs, surgical case delay, and differences in gastric volume by type of GLP-1 agonist. Occurrence of a full stomach was evaluated using a propensity weighted generalized estimating equation model with a logit link and including random physician effects to account for correlation between ultrasound volume measurements collected by the same physician. Propensity weights were used to account for potential confounder and heterogeneity between participants taking versus not taking GLP-1 agonists and were calculated using inverse probability treatment weighting from a logistic regression model including patient age, biologic sex, self-reported race, BMI, and NPO time. Model assumptions were checked graphically. Differences in antral grade (0–2) between groups were evaluated using the Cochran Armitage trend test. Differences in surgical delay or aspiration rates between groups were evaluated using Fisher’s Exact tests. In patients taking GLP-1 agonists, differences in gastric volume by type of GLP-1 agonist (semaglutide, dulaglutide, other) were evaluated using a Kruskal Wallace test. In patients taking GLP-1 agonists, associations between gastric volume and last GLP-1 agonist dose were examined using Spearman’s rank correlation.

Variables in the data were missing between 0 and 5 % of observations. Multiple imputation with 10 imputations was used to impute missing values prior to all analyses. Results were reported based on the pooled estimates across imputations for analyses that included variables with missing values. Sensitivity analyses were conducted using only complete cases and results were compared to results from the imputed data. All analyses were conducted in SAS v. 9.4 (SAS Institute, Cary, NC, USA).

## 3. Results

**Fig. 2.**
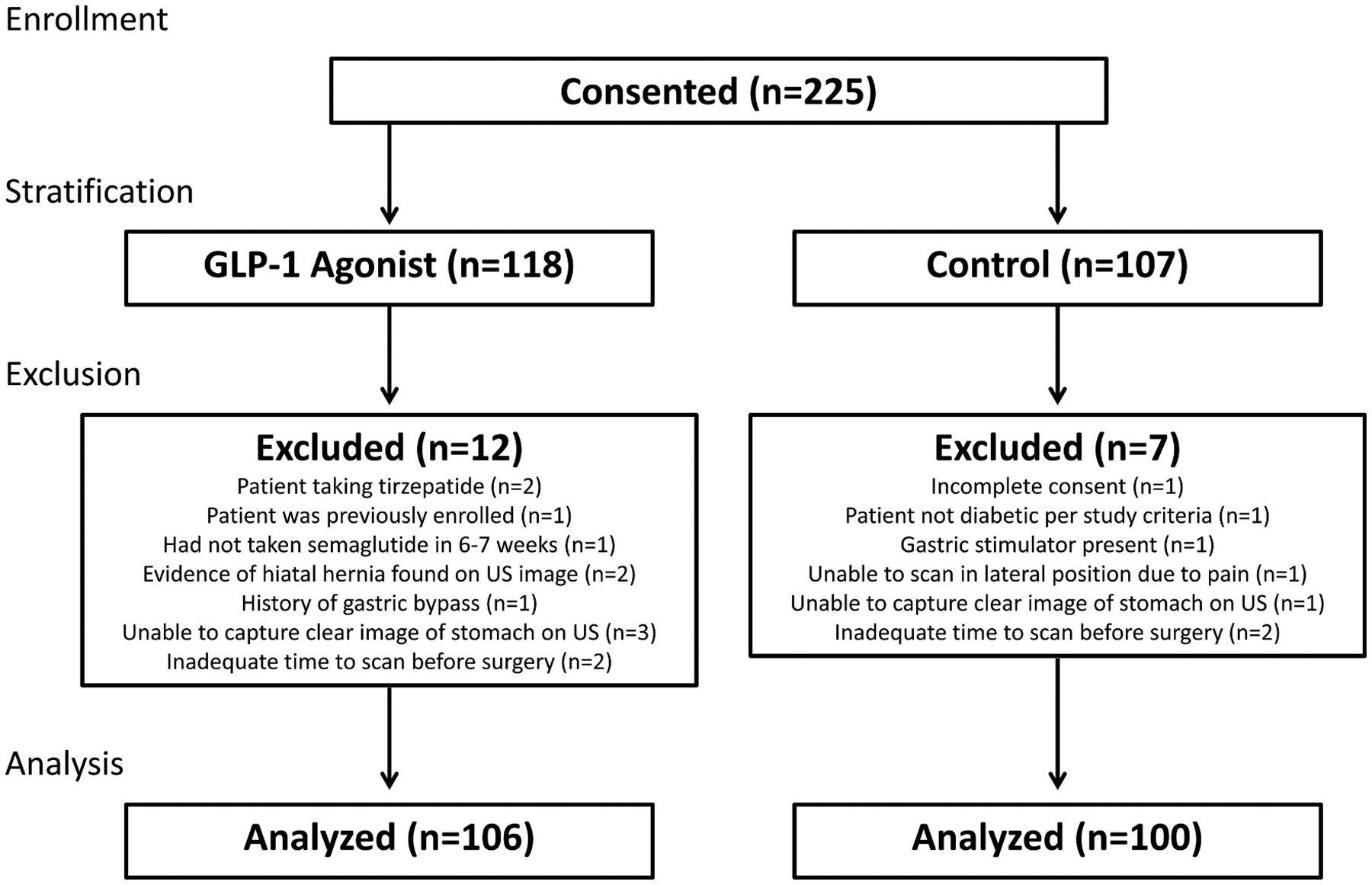
Patient enrollment flow diagram. GLP-1 agonist, glucagon-like peptide-1 agonists.

Between 8/9/2023 and 4/15/2024, 225 patients provided informed consent to participate. While 19 were excluded, 206 participants completed the study: 100 not taking a GLP-1 agonist and 106 taking a GLP-1 agonist (Fig. 2).

Patient characteristics are presented in Table 1. Subjects taking a GLP-1 agonist were younger with a higher BMI (*P* = 0.002 and *P* < 0.001, respectively). The groups were otherwise similar. Most GLP-1 agonists (96.23 %) were prescribed as weekly injections.

### 3.1. Gastric volume and ultrasound findings

The median (IQR; range) gastric volume in those not on a GLP-1 agonist was 0.16 mL/kg (0.44; 0 to 2.97) compared to 0.61 mL/kg (0.98; 0 to 2.85) in those taking a GLP-1 agonist (*P* < 0.001). In the multivariable logistic-log-normal mixed model controlling for patient age, BMI, and fasting duration, GLP1 use was associated with twice the odds of at least some stomach content (*P* = 0.022; OR (95 % CI): 1.98 (1.10, 3.54)); furthermore, given that some gastric volume was observed, those taking a GLP-1 agonist had 75 % greater volume on average compared to those not on a GLP-1 agonist (*P* < 0.001; mean % difference (95 % CI): 76.0 (41.4, 119.0); Supplemental Table 1).

In the propensity weighted model of gastric volume, GLP-1 agonist use had over 11 times the odds of a full stomach compared with patients not taking a GLP-1 agonist [*P* < 0.0001; OR (95 %) 11.3 (5.2, 24.7)]. Increased Perlas grade was found with GLP-1 agonist usage (P < 0.001; Table 2).

### 3.2. Delays and aspiration

Of the 206 participants, 196 proceeded with the scheduled surgery without delay. Participants taking a GLP-1 agonist (*n* = 6; 5.6 %) were more likely to require a surgical delay or cancelation compared to those not taking a GLP-1 agonist (*n* = 0; 0 %; *P* = 0.029). One participant (0.94 %) taking a GLP-1 agonist experienced an aspiration event. This subject had a preoperative gastric volume measuring 0.56 mL/kg of clear liquids.

### 3.3. Gastric volume by GLP-1 agonist type, duration, and cessation

**Fig. 3.**
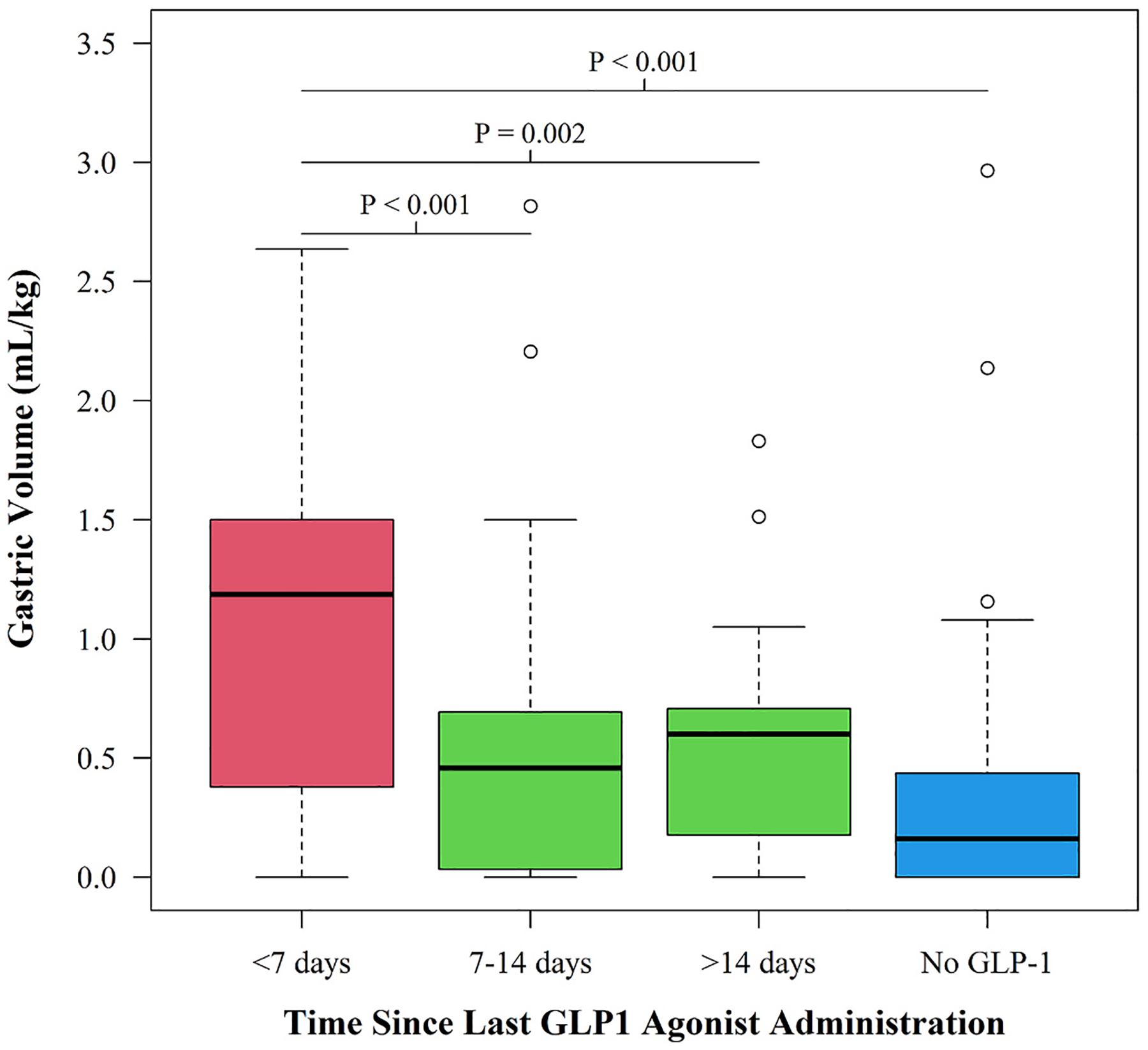
Boxplot of gastric volume by GLP-1 status: No GLP-1 use, last GLP-1 dose to surgery >14 days, between 7 and 14 days, and < 7 days. *P*-values are Bonferroni corrected for 6 pairwise comparisons.

As a secondary analysis, we also examined associations between gastric volume with class of GLP-1 agonist, duration of time on GLP-1 agonist, and time from last dose of GLP-1 agonist (semaglutide, dulaglutide, other) and gastric volume measurement among the subset of participants on GLP-1 agonist. There were no notable differences in gastric volume between the different classes of GLP-1 agonists (*P* = 0.538). There was also not an association between gastric volume and duration of time on a GLP-1 agonist (*P* = 0.923). Participants who had their last GLP-1 dose within 7 days of surgery had significantly higher gastric volumes relative to those who had their last dose was between 7 and 14 days or those whose last dose was more than 14 days from time of surgery (*P* < 0.001 for both comparisons). However, there was not a difference in gastric volume between those whose last dose was between 7 and 14 days of surgery relative to those whose last dose was more than 14 days from surgery (*P* = 0.937). Fig. 3 shows the boxplot of gastric volume by time from last GLP-1 agonist dose. Patients taking a GLP-1 agonist less than 7 days prior to surgery had significantly higher gastric volumes compared to controls (no GLP-1, P < 0.001) or patients that had not taken a GLP-1 agonist in over 14 days (*P* = 0.002) or in the last 7–14 days (P < 0.001).

## 4. Discussion

This single center, prospective, observational cohort trial found a significant increase in the gastric volume of appropriately fasted diabetic patients on GLP-1 agonists when compared to fasted diabetic patients not on GLP-1 agonists. Moreover, diabetic patients on GLP-1 agonists had over 11 times the odds of having a full stomach in our propensity weighted model. There was notable heterogeneity between the no GLP-1 and GLP-1 groups in patient age, weight, and BMI. Use of propensity weights for GLP-1 usage does partially address this heterogeneity to improve generalizability of these findings. Our multivariable logistic-log-normal mixed model controlling for patient age, BMI, and fasting duration revealed a 75 % greater volume in patients on GLP-1 agonists. A comparable study of 220 patients revealed significant association between semaglutide use and increased residual gastric contents when semaglutide was taken within 10 days of the procedure (OR 36.97, 95 % CI 16.54–99.32) [22]. Similar results were seen in a prospective cross-sectional study of 124 patients which revealed a 30.5 % increase in residual gastric contents (RGC), defined as presence of solids, thick liquids, or more than 1.5 mL/kg of clear liquids on gastric ultrasonography, in patients taking GLP-1 agonists (adjusted prevalence ratio 2.48; 95 % CI 1.23–4.97) [23]. Likewise, in a prospective study of 20 volunteers, semaglutide users were found to have solids present on gastric ultrasonography at higher rates than non-semaglutide users after an eight hour fast (RR 7.36, CI 1.13–47.7, *P* = 0.005) [24]. Similar findings have also been noted in retrospective studies of patients undergoing elective esophagogastroduodenoscopy. In a large retrospective study consisting of 35,183 patients undergoing esophagogastroduodenoscopy of which 922 took GLP-1 agonists, GLP-1 agonist users were four times more likely to have RGC (OR 4.08, *P* < 0.0001) [25]. In the same way, smaller retrospective studies revealed increased RGC in GLP-1 agonist users, with a prevalence ratio of 5.15 (CI 1.92–12.92) and OR 11.57 (CI 1.48–90.44) [26,27]. Our study is the largest prospective study describing gastric volume on ultrasonography in patients on and off GLP-1 agonist therapy with drug discontinuation times over two weeks and contributes to the growing knowledge in this area after our own experience with adverse intraoperative events in this patient population [8].

Given increased concerns for increased aspiration risk in patients on GLP-1 agonist therapy, initial ASA consensus-based guidelines recommend holding weekly dosed GLP-1 agonists for at least one week prior to elective surgery [11]. Although more recent publications [12–15] recommended GLP-1 agonists may be continued perioperatively, they emphasize an individualized, multidisciplinary, shared decision-making process [12–15]. They further state that an increased risk for delayed gastric emptying should prompt providers to consider holding GLP-1 agonists consistent with the original ASA guidance [12] versus three half-lives of the medication [14] versus GLP-1 agonist dose de-escalation [13]. Risk factors listed include escalation of GLP-1 agonist dose, higher doses of GLP-1 agonists, weekly dosing, presence of gastrointestinal symptoms, and other medical conditions that may delay gastric emptying [12]. As our secondary analysis of only patients on GLP-1 agonists noted that patients who held their weekly dose less than seven days prior to surgery had significantly higher gastric volumes compared with subjects that held their GLP-1 agonists for seven or greater days, our data suggests that holding GLP-1 agonist therapy for 7 days decreases gastric volumes. Notably, 96.33 % of our patients were on weekly GLP-1 injection, which fall into the increased risk category described above. It is further remarkable that there was no difference observed in the gastric volumes of GLP-1 agonist patients that held their dose for 7–14 days versus greater than 14 days. This contrasts with results found by two recent studies which found no association between duration of GLP-1 agonist discontinuation and odds of increased RGC (OR 0.86, CI 0.65–1.14) [23], (OR 0.76, CI 0.26–2.06) [22,23]. However, the majority of their populations took their last dose within 10 days of surgery so there is no data for patients holding longer than 10 days.

Given that patients on GLP-1 agonists had over 11 times the odds of having a full stomach, additional interventions, such as a clear liquid diet for 24 h prior to surgery, preoperative gastric POCUS, and use of rapid sequence induction may have utility in this population and has been in guidance statements from multiple societies and in expert opinions [12–15]. Our data conflicts with the clinical practice recommendation by Milder et al. [14] that stated GLP-1 agonists should be held for at least three half-lives (20 days for weekly-dosed subcutaneous semaglutide) if it is deemed necessary to discontinue the drug perioperatively. Our results suggest that there may not be utility in holding weekly-dosed GLP-1 agonist therapy for greater than seven days and suggest that the initial ASA consensus-based guidelines may be adequate for discontinuation of weekly-dosed GLP-1 agonists prior to surgery [11]. This also conflicts with recent consensus statements that allow for continuation of GLP-1 therapy through the perioperative period [12–15].

Our study design and methodology are very generalizable. Gastric ultrasound is a relatively simple perioperative ultrasound technique. The implications of higher gastric volumes in fasted patients underscores the importance of preoperative assessment of gastric volumes in patients taking GLP-1 agonists as they may be at a higher risk of regurgitation of gastric contents on induction or emergence of anesthesia.

### 4.1. Study limitations

Our study has some limitations. First, it was an observational study which can be prone to bias and confounding making it difficult to draw causation. As perioperative personnel learned more about GLP-1 agonists and concerns for aspiration, many patients received gastric ultrasounds during the study time period that were not consented for the study and captured in our data. Similarly, we excluded patients that were not diabetic but were on GLP-1 agonists for weight loss or “pre-diabetes”. Limiting our patients to diabetics was done since arguments had been raised that this was a diabetic issue and not a GLP-1 agonist issue by both our surgical and anesthesia colleagues. However, a recent publication demonstrated no difference in gastric volumes between diabetic and non-diabetic patients [28]. Thus, future studies should include patients on GLP-1 agonists regardless of diabetic status. We also excluded patients on tirzepatide as it is an agonist for both GLP-1 and glucose-dependent insulinotropic polypeptide receptors. Future studies should examine if tirzepatide has an increased or similar impact on gastric volume compared to GLP-1 agonists. Additionally, while our study revealed increased rates of surgery delay and cancelation in patients on GLP-1 agonist, we did not capture if our gastric ultrasound findings changed anesthetic management for patients that did have increased gastric contents less than 1.5 mL/kg (e.g, change from monitored anesthesia care to rapid sequence intubation), and we were not able to definitively verify this on retrospective review. This should be examined in the future. Finally, one patient taking a GLP-1 agonist was reported to have a perioperative aspiration. This patient had a gastric volume of 0.56 mL/kg (129.9 kg, 182.9 cm, ideal body weight 78 kg) that appeared hypoechoic, consistent with clear liquids, on gastric ultrasound. Intraoperatively, the patient had bilious emesis during neuraxial anesthesia and monitored anesthesia care with no evidence of solid or particulate contents. Oral pharyngeal suctioning and decreased sedation was initiated, and the patient protected their own airway without requiring intubation. The last dose of semaglutide was 11 days prior to surgery. While some have argued for a more conservative upper limit of normal for fasting gastric volumes at 0.8 mL/kg [29], our patient would have still remained below this threshold. Additionally, a recent meta-analyses of 12 studies with 1203 subjects found a more liberal upper level of normal for gastric volume (median 0.6 mL/kg with 2.3 mL/kg as the 95th percentile) [30], and our patient would have fallen below these numbers as well. More studies are likely needed to elucidate an agreed upon upper level of normal for gastric volume and whether qualitative (e.g. Perlas grade) versus quantitative assessment of gastric volume is more clinically important in assessing risk of aspiration.

### 4.2. Conclusion

In summary, this observational trial found diabetic patients taking GLP-1 agonists to have a significant increase in gastric volume and over 11 times the odds of having a full stomach compared to diabetic patients not on GLP-1 agonists. Gastric volumes were also significantly higher in patients who had taken their GLP-1 agonists in the last seven days compared to those who had held their GLP-1 agonist therapy for a week or more. Future studies should investigate the impact of a preoperative clear liquid diet on gastric volume in patients take GLP-1 agonists, include patient taking GLP-1 for weight loss regardless of diabetic status, and describe the impact of tirzepatide on perioperative gastric volumes. These findings have a large impact on the direction for preoperative counseling on the administration of these medications as well as a profound impact on preanesthetic planning.

## Supplementary Material

Table S1

## Figures and Tables

**Table 1:** Participant characteristics by GLP-1 agonist status.

	None (*N* = 100)	GLP-1 (*N* = 106)	*P*
Age, years, mean ± SD	66.2 ± 11.7	61.5 ± 10.1	0.002
Height, cm, mean ± SD	172.2 ± 11.6	171.1 ± 9.59	0.489
Weight, kg, mean (SD)	88.1 ± 19.5	94.2 ± 17.8	0.019
BMI, kg/m^2^, mean (SD)	29.6 ± 4.96	32.0 ± 4.40	<0.001
Male sex, n (%)	55 (55.0)	48 (45.3)	0.163
Race, n (%)			0.447
Black	39 (39.0)	37 (34.9)	
White	58 (58.0)	62 (58.5)	
Other	3 (3.00)	7 (6.60)	
Ethnicity			0.288
Hispanic	3 (3.00)	0 (0.00)	
Non-Hispanic	95 (95.0)	104 (98.1)	
Other	2 (2.00)	2 (1.89)	
Diabetes Type II, n (%)	97 (97.0)	106 (100.0)	0.113
Diabetes duration, years, median (IQR; range)	10 (16; 0, 61)	10 (14; 0, 57)	0.436
Gastroparesis, n (%)	2 (2.00)	2 (1.89)	1.000
Chronic kidney disease, n (%)	22 (22.0)	16 (15.1)	0.202
Severe nausea and vomiting, n (%)	1 (1.00)	3 (2.83)	0.622
Abdominal bloating, n (%)	2 (2.00)	3 (2.83)	1.000
Abdominal pain, n (%)	1 (1.00)	2 (1.89)	1.000
Fasting duration, hours, mean ± SD			
Solids	16.3 ± 4.66	17.3 ± 7.94	0.296
Clear liquids	14.9 ± 5.71	13.9 ± 5.21	0.202
Type of GLP-1 agonist, n (%)			
Dulaglutide (Trulicity)	–	37 (34.6)	
Semaglutide (Ozempic)	–	61 (57.5)	
Semaglutide (Rybelsus)*	–	4 (3.77)	
Liraglutide (Victoza, Saxenda)	–	5 (4.72)	
Duration GLP-1, days, median (IQR; range)	–	375 (534; 1, 4250)	
Time since last GLP1 dose, days, mean ± SD	–	10.9 ± 5.26	

BMI, body mass index; GLP-1, glucagon-like peptide 1 agonist; *, all GLP-1 agonists were prescribed as weekly injections except Semaglutide (Rybelsus) which was taken by mouth daily; –, not applicable (no data in the none group).

**Table 2:** Gastric ultrasound findings.

	No GLP-1 (N = 100)	GLP-1 (N = 106)	*P*
Gastric volume (ml/kg), median (IQR)	0.16 (0.44)	0.61 (0.98)	< 0.001
Perlas Grade, n (%)			<0.001
0	66 (66.0)	33 (31.1)	
1	32 (32.0)	41 (38.7)	
2	2 (2.00)	32 (30.2)	

GLP-1, glucagon-like peptide 1 agonist.

## HIGHLIGHTS

- GLP-1 agonist therapy was associated with higher residual gastric volumes.
- Higher gastric volumes seen with cessation of GLP-1 agonists within 7 days of surgery.
- No difference in gastric volumes when holding GLP-1 agonists 7–14 days and > 14 days.
- This opposes consensus-based guidelines on preoperative cessation of GLP-1 agonists.

## Appendix A. Supplementary data

Supplementary data to this article can be found online at https://doi.org/10.1016/j.jclinane.2025.111853.

## Glossary of terms

BMI: body mass index
CI: confidence intervals
CSA: cross-sectional area
GLP-1: Glucagon-like peptide-1
GV: gastric volume
IRB: institutional review board
IQR: interquartile range
