# Supplementary material for: Ultrasound assessment of preoperative gastric volume in fasted diabetic surgical patients: A prospective observational cohort study on the effects of glucagon-like peptide-1 agonists on gastric emptying
Source: J Clin Anesth. Author manuscript; Available in PMC 2026 Jan 13. (PMC12797078; doi:10.1016/j.jclinane.2025.111853)
Supplement: Table S1 [file NIHMS2134504-supplement-Table_S1.docx]

**Supplemental Table 1**: Two-stage logistic-log-normal mixed model of gastric volume. The logistic model estimates the probability a participant has a non-zero volume and in the second stage, conditional on having non-zero volume the log-normal model estimates the estimates the natural log of the volume. Values for the logistic model from stage 1 are reported as odds ratios (95% CI) for having a non-zero volume and values for the log-normal model for stage 2 are reported as the mean percent difference (95% CI) in gastric volume.

| **Logistic Model of Probability of Non-Zero Volume** | **Odds Ratio (95% CI)** | **P** |
| --- | --- | --- |
| GLP1, Yes vs. No | 1.98 (1.10, 3.54) | 0.022 |
| **Conditional Log-Normal Model of Volume** | **Mean % Difference in Volume (95% CI)** | **P** |
| GLP1, Yes vs. No | 76.0 (41.4, 119.0) | <0.001 |
